# Supplementary material for: Forest cover mediates large and medium-sized mammal occurrence in a critical link of the Mesoamerican Biological Corridor
Source: PLoS One. 2021 Mar 23;16(3):e0249072. doi: 10.1371/journal.pone.0249072 (PMC7996086; doi:10.1371/journal.pone.0249072)
Supplement: S1 File — Barbilla-Destierro Biological Corridor (Corridor) and portions of Central Volcanic Cordillera (CVC) and Talamanca-Cordillera Central (TC) Jaguar Conservation Units (JCUs), surveyed with camera traps from 2013–2017. (DOCX) [file pone.0249072.s001.docx]

### S1 File: Additional information on covariates selected *a priori* as being thought to have an influence on habitat use probability of medium and large mammals. Barbilla-Destierro Biological Corridor (Corridor) and portions of Central Volcanic Cordillera (CVC) and Talamanca-Cordillera Central (TC) Jaguar Conservation Units (JCUs), surveyed with camera traps from 2013-2017.

We calculated and standardized (1) site covariates in each 16 km^2^ grid cell using Arc Map 10.3.1 (ESRI^®^), QGIS 3.4.4 (Creative Commons Attribution-ShareAlike 3.0 license) and R (R Core Team 2015®; version 3.4.3) (Table S1). Mean EVI values (2) were derived from the study period dates for each block. Survey periods were Oct 2013 - January 2014 (Corridor Block 1), January 2014 - May 2014 (Corridor Block 2), August 2014 to April 2015 (CVC JCU), and September 2016 to April 2017 (TC JCU). Values for percent forest version 1 (forest v1;(3)) were specific to the year surveyed (Corridor Block 1: 2013, Corridor Block 2: 2014, CVC JCU: 2015, TC JCU: 2017), and became a binary layer such that forest comprised cells with > 30% forest cover. Forest version 2 (forest v2) was a product specific to Costa Rica (4), with JAXA land use classification using RADAR images implemented for areas with clouds. We created the distance layers (minimum distance to a primary road (4), minimum distance to a major settlement (main settlements in Provinces, Cantons and Districts excluding National Geographic Institute (IGN) exclusive areas) (4), minimum distance to any settlement (4), minimum distance to a strictly protected area (4), and minimum distance to a JCU (Panthera unpub. data)) by calculating the distance from the center point of each grid cell to the closest point, line or polygon in each of the layers. For the minimum distance to primary road covariate, only paved roads of one or more lanes were considered. Finally, human presence in each grid cell was the number of human detections per 1,000 trap nights in the camera trap stations, in which each event was separated by at least one hour at a given station. To calculate Effort, we standardized the sum of all trap nights on each occasion for every grid cell.

Table S1. Information of covariates selected *a priori* as being thought to have an influence on habitat use probability of medium and large mammals in the Barbilla-Destierro Biological Corridor (Corridor) and portions of Central Volcanic Cordillera (CVC) and Talamanca-Cordillera Central (TC) Jaguar Conservation Units (JCUs), surveyed with camera traps from 2013-2017.

| **Covariate** | **Source** | | **Resolution** | **Year** | | **Hypothesized relationship**  **to mammal habitat use** | |
| --- | --- | --- | --- | --- | --- | --- | --- |
| Mean EVI (Enhanced Vegetation Index) | MOD13Q1 – 16 Day | | 250 m | 2013, 2014, 2015, 2016, 2017 | | + | |
| Percent forest version 1 (forest v1) | Hansen et al. 2013 | | 30 m | CORRIDOR- Block1:2013, CORRIDOR-Block2: 2014, CVC: 2015, Talamanca: 2017 | | + | |
| Percent forest version 2 (forest v2) | Costa Rican National Forestry Inventory-SIREFOR | | 30 m | 2012 | | + | |
| Mean Elevation | SRTM DEM | | 30 m | NA | | - | |
| Mean Ruggedness | SRTM DEM | | 30 m | NA | | - | |
| Minimum distance to a primary road | Costa Rican National Geographic Institute (IGN) | | scale 1:25,000 | 2005 | | + | |
| Minimum distance to a major settlement | IGN and National Institute of Statistics and Census (INEC) | | scale 1:25,000 | 2013 | | + | |
| Minimum distance to any settlement | IGN and INEC. | | scale 1:25,000 | 2013 | | + | |
| Minimum distance to a strictly-protected area | CENIGA, IUCN Ia & II categories | | scale 1:50,000 | 2011 | | - | |
| Minimum distance to JCU | Panthera unpub. data | | 250 m | 2017 | | - | |
| Human presence | This study | | NA | 2013-2017 | | - | |
|  |  |  | | |  | |  |

Table S2. Correlation of site covariates selected *a priori* as being thought to have an influence on habitat use probability of jaguars and pumas in the Barbilla-Destierro Biological Corridor (Corridor) and portions of Central Volcanic Cordillera (CVC) and Talamanca-Cordillera Central (TC) Jaguar Conservation Units (JCUs), surveyed with camera traps from 2013-2017. Covariates with a pairwise correlation > 0.6 were not included in the same model


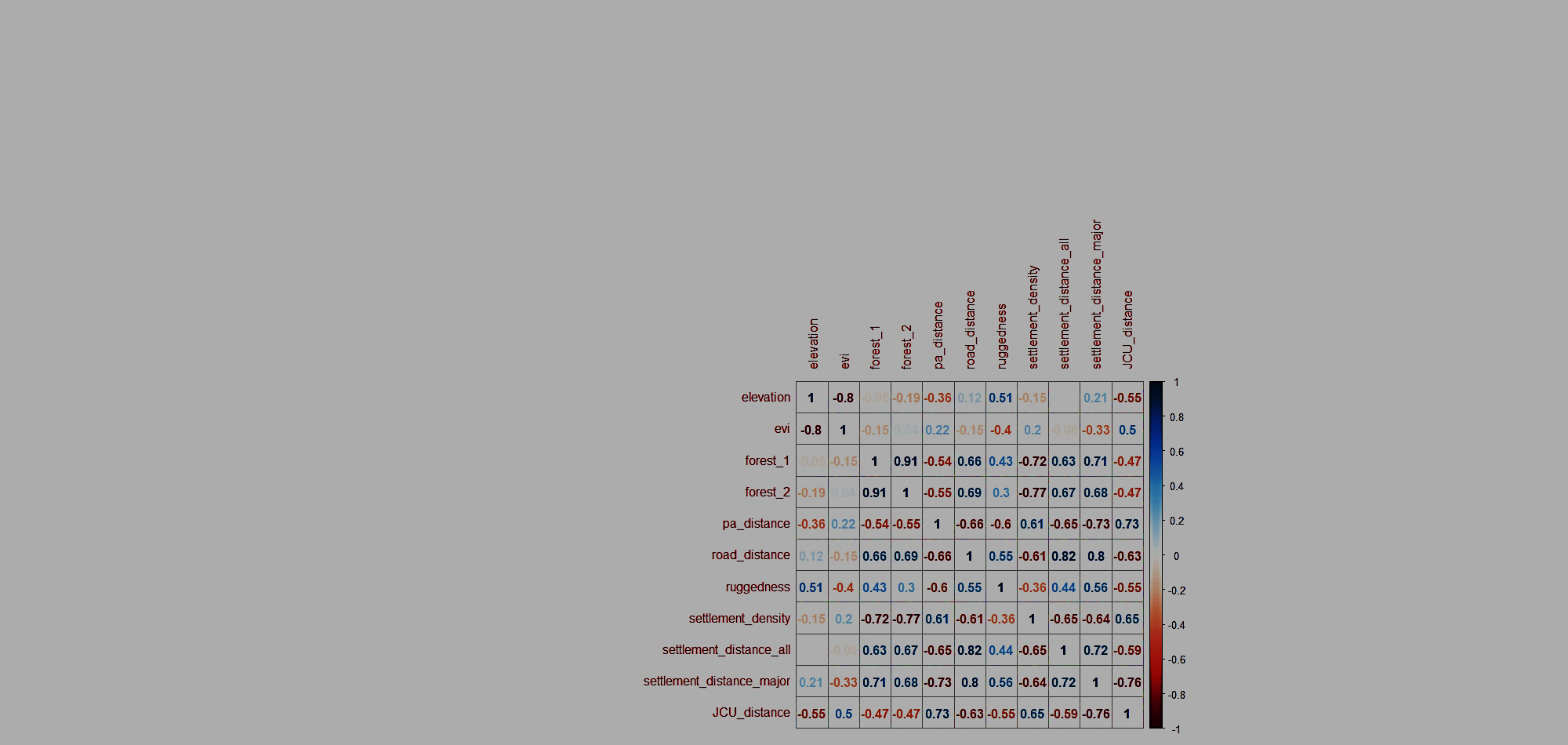


References for S1:

1. Schielzeth H. Simple means to improve the interpretability of regression coefficients. Methods Ecol Evol. 2010;1(2):103–13.

2. Didan K. MOD13Q1 MODIS/Terra vegetation indices 16-day L3 global 250m SIN grid V006 [Data set] [Internet]. 2015 [cited 2019 Jan 10]. Available from: https://doi.org/10.5067/MODIS/MOD13Q1.006

3. Hansen MC, Potapov P V., Moore R, Hancher M, Turubanova SA, Tyukavina A, et al. High-resolution global maps of 21st-century forest cover change. Science (80- ). 2013;342(6160):850–3.

4. SINAC. Estado de conservación del jaguar (Panthera onca) en costa rica a través de la integración de datos de registro de la especie y modelaje del habitat idóneo. Heredia, Costa Rica; 2018.
