# Supplementary material for: Forest cover mediates large and medium-sized mammal occurrence in a critical link of the Mesoamerican Biological Corridor
Source: PLoS One. 2021 Mar 23;16(3):e0249072. doi: 10.1371/journal.pone.0249072 (PMC7996086; doi:10.1371/journal.pone.0249072)
Supplement: S3 File — Barbilla-Destierro Biological Corridor (Corridor) and portions of Central Volcanic Cordillera (CVC) and Talamanca-Cordillera Central (TC) Jaguar Conservation Units (JCUs), surveyed with camera traps from 2013–2017. (DOCX) [file pone.0249072.s003.docx]

### S3 File: Additional description on literature review conducted to select species included as prey species for jaguars (*Panthera onca*) and pumas (*Puma concolor*). Barbilla-Destierro Biological Corridor (Corridor) and portions of Central Volcanic Cordillera (CVC) and Talamanca-Cordillera Central (TC) Jaguar Conservation Units (JCUs), surveyed with camera traps from 2013-2017.

We found and reviewed 23 studies, including peer-reviewed publications (*N*=19) and theses (*N*=4), describing jaguar and puma prey species from Mexico to Panama (Table S4). In total, there were four large prey species for both jaguars and pumas, ten medium prey species for jaguars and 11 medium prey species for (Tables S4). We included species as medium or large terrestrial mammal prey if the species or genus were mentioned in more than one document. The species that were not mentioned or that were mentioned only in one document were excluded, as they were considered opportunistic cases and thus probably not having considerable effect on jaguar or puma habitat use. Species mentioned in one document but not considered for this analysis were fox, coyote, ocelot and tayra for jaguar, and coyote for puma. Species that were not mentioned as prey for jaguar and puma were northern-naked-tailed armadillo, jaguarundi, margay, oncilla, and tapir. Additionally, puma was not reported as prey for jaguars, and grison, jaguar and ocelot were not reported in the puma diet in any of the documents. We included domestic pig as part of jaguar and puma diet given that they could represent an important prey item for jaguars and pumas in the study area. This is especially true in or near the indigenous territories present in the east side of the Corridor, where domestic pigs are feral and roam freely, were frequently detected by the camera traps, and were predated by both species on several occasions (before and during this study; R. Salom-Perez*, personal observation*). Other domestic animals or arboreal species were not considered in this analysis because we considered that camera traps (or their location) were not ideal for detecting these species.

Table S4. List of species or genus found in the literature review of 23 investigations related to diet of jaguars (*Panthera onca*) or pumas (*Puma concolor*) from Mexico to Panama. In gray are species or genus detected in the present study. *Included for the estimate of prey richness for jaguar. + Included for the estimate of prey richness for puma.

| **Class** | **Order** | **Family** | **Genus** | **Species** | **Note** | **Citations as jaguar diet** | **Citations as puma diet** |
| --- | --- | --- | --- | --- | --- | --- | --- |
| Aves | Galliformes | Cracidae | *Crax* | *rubra* |  | 2; 3; 15 | 21 |
| Aves | Galliformes | Cracidae | *Ortalis* | *poliocephala* |  |  | 12 |
| Aves | Galliformes | Cracidae | *Ortalis* | *vetula* |  | 21 | 21 |
| Aves | Galliformes | Phasianidae | *Agriocharis* | *ocellata* |  | 13; 23 |  |
| Aves | Galliformes | Phasianidae | *Gallus* | *gallus* | Domestic animal | 1 |  |
| Aves | NA | NA | NA | NA | Bird | 1; 2; 5; 6; 18; 23 | 5; 6; 8; 10; 18 |
| Malacostraca | Decapoda | NA | NA | NA |  |  | 14 |
| Mammalia | Artiodactyla | Bovidae | *Bos* | *taurus* | Domestic animal | 14 |  |
| Mammalia | Artiodactyla | Bovidae | *Capra* | *hircus* | Domestic animal | 22 | 12; 19; 22 |
| Mammalia | Artiodactyla | Bovidae | NA | NA | Domestic animal |  | 12; 18 |
| Mammalia | Artiodactyla | Bovidae | *Ovis* | *aries* | Domestic animal | 14 |  |
| Mammalia | Artiodactyla | Cervidae | *Mazama* | sp. |  | 6; 13 | 6; 13 |
| Mammalia | Artiodactyla | Cervidae | *Mazama* | *temama*+* |  | 1; 3; 7; 14; 18; 21; 23 | 3; 8; 10; 14; 18; 21 |
| Mammalia | Artiodactyla | Cervidae | NA | NA |  | 2; 6 | 6 |
| Mammalia | Artiodactyla | Cervidae | *Odocoileus* | *virginianus*+* |  | 5; 6; 13; 15; 21; 22 | 4; 5; 6; 12; 13; 14; 15; 18; 21; 22 |
| Mammalia | Artiodactyla | Cetacea | NA | NA | Marine dolphin | 17 |  |
| Mammalia | Artiodactyla | Suidae | *Sus* | *scrofa*+* | Domestic animal |  | 19 |
| Mammalia | Artiodactyla | Tayassuidae | *Pecari* | *tajacu*+* |  | 2; 4; 5; 6; 7; 9; 10; 13; 14; 15; 18; 21 | 4; 5; 6; 8; 10; 13; 14; 15; 18; 19; 21 |
| Mammalia | Artiodactyla | Tayassuidae | *Tayassu* | *pecari* |  | 1; 3; 6; 7; 10; 13; 14; 15; 23 | 6; 13; 14 |
| Mammalia | Carnivora | Canidae | *Canis* | *familiaris* | Domestic animal | 14; 21 |  |
| Mammalia | Carnivora | Canidae | *Canis* | *latrans* |  | 15 | 21 |
| Mammalia | Carnivora | Canidae | *Canis* | sp. | Unclear if domestic or wild | 13 |  |
| Mammalia | Carnivora | Canidae | *Cerdocyon* | *thous* |  | 10 |  |
| Mammalia | Carnivora | Canidae | *Urocyon* | *cinereoargenteus+* |  | 14 | 6; 12; 18; 21 |
| Mammalia | Carnivora | Felidae | *Leopardus* | *pardalis* |  | 3 |  |
| Mammalia | Carnivora | Felidae | NA | NA | *Panthera onca or Puma concolor* | 23 |  |
| Mammalia | Carnivora | Mephitidae | *Conepatus* | *leuconotus* |  | 22 |  |
| Mammalia | Carnivora | Mephitidae | *Conepatus* | *mesoleucus* |  |  | 12 |
| Mammalia | Carnivora | Mephitidae | *Conepatus* | *semistriatus*+* |  | 13 |  |
| Mammalia | Carnivora | Mephitidae | *Conepatus* | sp. |  | 14; 18 | 21 |
| Mammalia | Carnivora | Mephitidae | NA | NA | *Spilogale putorius or Conepatus semistriatus* | 1 |  |
| Mammalia | Carnivora | Mephitidae | *Spilogale* | *putorius* |  |  | 12 |
| Mammalia | Carnivora | Mustelidae | *Eira* | *barbara+* |  | 13 | 13; 18 |
| Mammalia | Carnivora | Mustelidae | *Galictis* | *vittata** |  | 14; 18 |  |
| Mammalia | Carnivora | Mustelidae | *Mustela* | *frenata* |  |  | 12 |
| Mammalia | Carnivora | Procyonidae | *Bassariscus* | *astutus* |  |  | 12 |
| Mammalia | Carnivora | Procyonidae | *Bassariscus* | *sumichrasti* |  |  | 13 |
| Mammalia | Carnivora | Procyonidae | *Nasua* | *narica*+* |  | 1; 2; 5; 6; 7; 9; 13; 14; 18; 21; 22 | 5; 6; 8; 11; 12; 13; 18; 19; 21; 22 |
| Mammalia | Carnivora | Procyonidae | *Potos* | *flavus* |  | 1; 6; 7; 13; 14; 18; 23 | 6; 13; 14; 18 |
| Mammalia | Carnivora | Procyonidae | *Procyon* | *lotor*+* |  | 14; 18; 22 | 12; 21; 22 |
| Mammalia | Cyngulata | Dasypodidae | *Dasypus* | *novemcinctus*+* |  | 1; 2; 5; 6; 7; 13; 14; 18; 21; 22; 23 | 4; 5; 6; 8; 10; 12; 13; 14; 15; 18; 22 |
| Mammalia | Didelphimorphia | Didelphidae | *Didelphis* | *marsupialis*+* |  | 1 | 8; 15 |
| Mammalia | Didelphimorphia | Didelphidae | *Didelphis* | sp. |  | 13; 14 |  |
| Mammalia | Didelphimorphia | Didelphidae | *Didelphis* | *virginiana* |  | 5; 21 | 5; 12; 15; 22 |
| Mammalia | Didelphimorphia | Didelphidae | *Marmosa* | *canescens* |  |  | 5 |
| Mammalia | Didelphimorphia | Didelphidae | *Philander* | *opossum* |  | 13; 23 | 8; 13 |
| Mammalia | Lagomorpha | Leporidae | *Sylvilagus* | *brasiliensis= gabbi*+* |  | 23 |  |
| Mammalia | Lagomorpha | Leporidae | *Sylvilagus* | *cuniculaurius* |  |  | 12; 22 |
| Mammalia | Lagomorpha | Leporidae | *Sylvilagus* | *floridanus* |  | 4; 15; 22 | 12; 22 |
| Mammalia | Lagomorpha | Leporidae | *Sylvilagus* | sp. |  | 13 | 13; 21; 22 |
| Mammalia | NA | NA | NA | NA | Small mammal |  | 8 |
| Mammalia | Perissodactyla | Equidae | *Equus* | sp. | Domestic animal | 21 |  |
| Mammalia | Pilosa | Bradypodidae | *Bradypus* | variegatus |  | 10 | 8; 10 |
| Mammalia | Pilosa | Bradypodidae | NA | NA |  | 23 |  |
| Mammalia | Pilosa | Choloepodidae | *Choloepus* | *hoffmanni* |  | 3 | 8; 10 |
| Mammalia | Pilosa | Myrmecophagidae | *Tamandua* | *mexicana*+* |  | 1; 2; 14; 18 | 8; 10; 13 |
| Mammalia | Primates | Atelidae | *Alouatta* | *palliata* |  |  | 3; 19 |
| Mammalia | Primates | Atelidae | *Ateles* | *geoffroyi* |  | 13; 15; 23 | 3 |
| Mammalia | Primates | Cebidae | *Cebus* | *imitator* |  | 3 | 3; 8 |
| Mammalia | Primates | NA | NA | NA |  | 6; 10 | 6; 10 |
| Mammalia | Rodentia | Cricetidae | *Ototylomis* | *phyllotis* |  |  | 13 |
| Mammalia | Rodentia | Cricetidae | *Peromyscus* | *yucatanicus* |  | 13 | 13 |
| Mammalia | Rodentia | Cuniculidae | *Cuniculus* | *paca*+* |  | 2; 6; 7; 13; 14; 18; 23 | 6; 8; 10; 13; 14; 18 |
| Mammalia | Rodentia | Dasyproctidae | *Dasyprocta* | *punctata*+* |  | 6; 9; 10; 13; 18 | 3; 6; 8; 10; 13 |
| Mammalia | Rodentia | Echimyidae | *Proechimys* | *semispinosus* |  |  | 3; 8 |
| Mammalia | Rodentia | Erethizontidae | *Sphiggurus* | *mexicanus* |  |  | 3; 6; 14 |
| Mammalia | Rodentia | Heteromyidae | *Heteromys* | *desmarestianus* |  |  | 13 |
| Mammalia | Rodentia | Heteromyidae | *Heteromys* | sp. |  | 14 |  |
| Mammalia | Rodentia | Heteromyidae | *Lyomis* | sp. |  |  | 12 |
| Mammalia | Rodentia | NA | NA | NA |  | 1 | 5; 8 |
| Mammalia | Rodentia | Sciuridae | *Sciurus* | *aureogaster* |  |  | 12 |
| Mammalia | Rodentia | Sciuridae | *Sciurus* | *granatensis* |  |  | 8 |
| Reptilia | Crocodilia | Crocodylidae | *Crocodylus* | sp. |  | 13 |  |
| Reptilia | NA | NA | NA | NA |  | 1; 10 |  |
| Reptilia | Squamata | Iguanidae | *Ctenosaura* | *pectinata* |  | 5; 22 | 5; 22 |
| Reptilia | Squamata | Iguanidae | *Ctenosaura* | *similis* |  |  | 15 |
| Reptilia | Squamata | Iguanidae | *Iguana* | *iguana* |  | 3; 14; 18; 23 | 8; 14 |
| Reptilia | Squamata | NA | NA | NA | Snake | 1; 2 | 5; 8; 14 |
| Reptilia | Squamata | Teiidae | *Cnemidophorus* | sp. |  |  | 13 |
| Reptilia | Testudines | Cheloniidae | *Chelonia* | *mydas* |  | 20; 23 |  |
| Reptilia | Testudines | Cheloniidae | *Lepidochelys* | *olivacea* |  | 3; 15 |  |
| Reptilia | Testudines | Geoemydidae | *Rhinoclemmys* | *areolata* |  | 13 |  |
| Reptilia | Testudines | Kinosternidae | *Kinosternon* | *integrum* |  |  | 12 |
| Reptilia | Testudines | Kinosternidae | *Staurotypus* | *triporcatus* |  | 15 |  |
| Reptilia | Testudines | NA | NA | NA | River turtle | 1; 2; 18 |  |
| *NA: | Not availble |  |  |  |  |  |  |

References for S3:

1. Rabinowitz AR, Nottingham Jr BG. Ecology and behavior of the jaguar in Belize, Central America. J Zool. 1986;210:149–59.

2. Aranda M. Importancia de los pecaries (*Tayassu* spp.) en la alimentación del jaguar (Panthera onca). Acta Zoológica Mex (nueva Ser. 1994;(62):11–22.

3. Chinchilla FA. La dieta del jaguar (*Panthera onca*), el puma (*Felis concolor*) y el manigordo (*Felis pardalis*) (Carnivora: Felidae) en el Parque Nacional Corcovado, Costa Rica. Rev Biol Trop. 1997;45(3):1223–9.

4. Farrell LE, Roman J, Sunquist ME. Dietary separation of sympatric carnivores identified by molecular analysis of scats. Mol Ecol. 2000;9(10):1583–90.

5. Núñez R, Miller B, Lindzey F. Food habits of jaguars and pumas in Jalisco, Mexico. J Zool. 2000;252(3):373–9.

6. Novack AJ, Main MB, Sunquist ME, Labisky RF. Foraging ecology of jaguar (*Panthera onca*) and puma (*Puma concolor*) in hunted and non-hunted sites within the Maya Biosphere Reserve, Guatemala. J Zool. 2005;267(2):167–78.

7. Weckel M, Giuliano W, Silver S. Jaguar (*Panthera onca*) feeding ecology: Distribution of predator and prey through time and space. J Zool. 2006;270(1):25–30.

8. Moreno RS, Kays RW, Samudio R. Competitive Release in diets of ocelot (*Leopardus pardalis*) and puma (*Puma concolor*) after jaguar (*Panthera onca*) decline. J Mammal. 2006;87(4):808–16.

9. Corrales-Gutiérrez D, Cardenal-Porras J. Ecología poblacional de jaguar (*Panthera onca*) y puma (*Puma concolor*) y dieta de jaguar, en el sector Pacífico de la Cordillera de Talamanca, Costa Rica. Universidad Latina de Costa Rica; 2008.

10. Moreno R. Informacion preliminar sobre la dieta de jaguares, y pumas en Cana, Parque Nacional Darien, Panama. Tecnociencia. 2008;10(1):115–26.

11. Bustamante A, Moreno R, Saenz JC. Predation of a coati (*Nasua narica*) by a puma (*Puma concolor*) in the southeast of the Osa Peninsula, Costa Rica. Acta Biológica Panamensis. 2009;1:39–45.

12. Monroy-Vilchis O, Gómez Y, Janczur M, Urios V. Food Niche of *Puma concolor* in Central Mexico . Wildlife Biol. 2009;15(1):97–105.

13. Chávez J. Ecologia y conservación del jaguar (*Panthera onca*) y puma (*Puma concolor*) en la región de Calakmul y sus implicaciones para la conservación de la península de Yucatán [Internet]. Universidad de Granada; 2010. Available from: http://digibug.ugr.es/handle/10481/5590

14. Foster RJ, Harmsen BJ, Valdes B, Pomilla C, Doncaster CP. Food habits of sympatric jaguars and pumas across a gradient of human disturbance. J Zool. 2010;280(3):309–18.

15. Montalvo Guadamuz VH. Cambios en la abundancia, actividad temporal y dieta de jaguar (*Panthera onca*), otros felinos y sus presas en el Parque Nacional Santa Rosa , Área de Conservación Guanacaste, Costa Rica. Universidad Nacional; 2012.

16. Castañeda FE, McCranie JR, Herrera LA. Natural History Notes: Staurotypus triporcatus predation. Herpetol Rev. 2013;(44):309.

17. Castañeda FE, Herrera LE, Pereira SC. Behaviour of two male jaguars scavenging on a marine dolphin in Honduras. Cat News. 2013;(58):11–2.

18. Figueroa O. The ecology and conservation of jaguars (*Panthera onca*) in central Belize: conservation status, diet, movement patterns and habitat use. University of Florida; 2013.

19. Bustamante A, Moreno R, Aliaga- Rossel E, Artavia A. Depredación del puma (*Puma concolor*) en un bosque Neotropical Centroamericano. Rev Latinoam Conserv. 2014;1(1):40–5.

20. Guilder J, Barca B, Arroyo-Arce S, Gramajo R, Salom-Pérez R. Jaguars (*Panthera onca*) increase kill utilization rates and share prey in response to seasonal fluctuations in nesting green turtle (Chelonia mydas mydas) abundance in Tortuguero National Park, Costa Rica. Mamm Biol. 2015;80(2):65–72.

21. Hernández-SaintMartín AD, Rosas-Rosas OC, Palacio-Núñez J, Tarango-Arambula LA, Clemente-Sánchez F, Hoogesteijn AL. Food Habits of Jaguar and Puma in a Protected Area and Adjacent Fragmented Landscape of Northeastern Mexico. Nat Areas J. 2015;35(2):308–17.

22. Gómez-Ortiz Y, Monroy-Vilchis O, Mendoza-Martínez GD. Feeding interactions in an assemblage of terrestrial carnivores in central Mexico. Zool Stud. 2015;54(JAN):16–23.

23. Arroyo-Arce S, Thomson I, Cutler K, Wilmott S. Feeding habits of the jaguar *Panthera onca* (Carnivora: Felidae) in Tortuguero National Park, Costa Rica. Rev Biol Trop. 2017;66(1):70–77.
