# Supplementary material for: Forest cover mediates large and medium-sized mammal occurrence in a critical link of the Mesoamerican Biological Corridor
Source: PLoS One. 2021 Mar 23;16(3):e0249072. doi: 10.1371/journal.pone.0249072 (PMC7996086; doi:10.1371/journal.pone.0249072)
Supplement: S4 File — Barbilla-Destierro Biological Corridor (Corridor) and portions of Central Volcanic Cordillera (CVC) and Talamanca-Cordillera Central (TC) Jaguar Conservation Units (JCUs), surveyed with camera traps from 2013–2017. (DOCX) [file pone.0249072.s004.docx]

### S4 File: Additional information on number of occupied cells, relative abundance and number of independent detections of medium and large-sized native mammals and domestic pig (n = 25). Barbilla-Destierro Biological Corridor (Corridor) and portions of Central Volcanic Cordillera (CVC) and Talamanca-Cordillera Central (TC) Jaguar Conservation Units (JCUs), surveyed with camera traps from 2013-2017.

Table S5. Number of occupied cells (*N=*63), relative abundance and number of independent detections of medium and large mammals and domestic pig (*N*=25) in the Barbilla-Destierro Biological Corridor (Corridor) and portions of Central Volcanic Cordillera (CVC) and Talamanca-Cordillera Central (TC) Jaguar Conservation Units (JCUs), surveyed with camera traps from 2013-2017. *Included for the estimate of prey richness for jaguar. + Included for the estimate of prey richness for puma.

|  |  | **Occupied cells** | | **Relative abundance (# of independent records/1000 trap nights)** | | | |  |
| --- | --- | --- | --- | --- | --- | --- | --- | --- |
| **Common name** | **Scientific name** | Mean | SD | Corridor | CVC JCU | TC JCU | Overall | **# of independent detections** |
| Agouti | *Dasyprocta punctata*^+^* | 41.28 | 1.39 | 32.95 | 16.02 | 49.81 | 33.90 | 573 |
| Nine Banded Armadillo | *Dasypus novemcinctus*^+^* | 56.27 | 1.04 | 33.62 | 27.47 | 3.14 | 24.31 | 411 |
| Ocelot | *Leopardus pardalis* | 54.14 | 1.38 | 24.13 | 10.01 | 25.35 | 21.53 | 364 |
| White Nosed Coati | *Nasua narica*^+^* | 54.73 | 1.96 | 22.12 | 11.44 | 8.53 | 16.33 | 276 |
| Coyote | *Canis latrans* | 25.12 | 1.67 | 16.76 | 14.31 | 0.00 | 11.83 | 200 |
| Common Opossum | *Didelphis marsupialis*^+^* | 42.35 | 2.41 | 8.60 | 20.89 | 6.28 | 10.53 | 178 |
| Tayra | *Eira barbara^+^* | 51.88 | 2.48 | 14.63 | 2.29 | 5.16 | 9.58 | 162 |
| Common Raccoon | *Procyon lotor*^+^* | 31.58 | 1.81 | 16.42 | 2.00 | 1.12 | 9.41 | 159 |
| Paca | *Cuniculus paca*^+^* | 24.92 | 2.60 | 1.45 | 0.29 | 14.58 | 4.67 | 79 |
| Puma | *Puma concolor* | 31.30 | 2.56 | 1.34 | 9.73 | 7.18 | 4.61 | 78 |
| Margay | *Leopardus wiedii* | 24.26 | 2.29 | 1.23 | 8.01 | 5.38 | 3.73 | 63 |
| Jaguarundi | *Puma yagouaroundi* | 44.44 | 4.92 | 3.91 | 2.58 | 4.04 | 3.67 | 62 |
| Jaguar | *Panthera onca* | 18.74 | 2.70 | 1.45 | 0.00 | 8.53 | 3.02 | 51 |
| Domestic Pig | *Sus scrofa*^+^* | 13.46 | 2.08 | 3.02 | 0.00 | 4.26 | 2.72 | 46 |
| Tapir | *Tapirus bairdii* | 9.14 | 0.97 | 0.34 | 8.87 | 1.79 | 2.48 | 42 |
| Tapeti Rabbit | *Sylvilagus brasiliensis*^+^* | 12.33 | 1.21 | 0.11 | 10.87 | 0.45 | 2.43 | 41 |
| Striped Hog Nosed Skunk | *Conepatus semistriatus*^+^* | 28.40 | 4.59 | 3.24 | 1.72 | 0.90 | 2.31 | 39 |
| Red Brocket Deer | *Mazama temama*^+^* | 22.77 | 3.27 | 0.56 | 4.29 | 2.92 | 1.95 | 33 |
| Collared Peccary | *Pecari tajacu*^+^* | 18.62 | 3.89 | 0.22 | 2.29 | 4.49 | 1.77 | 30 |
| Northern Tamandua | *Tamandua mexicana*^+^* | 39.12 | 7.87 | 1.90 | 0.57 | 0.22 | 1.18 | 20 |
| Nothern Naked Tailed Armadillo | *Cabassous centralis* | 7.06 | 2.64 | 1.34 | 0.00 | 0.45 | 0.83 | 14 |
| Greater Grison | *Gallictis vittata** | 13.38 | 4.98 | 0.89 | 0.57 | 0.22 | 0.65 | 11 |
| Oncilla | *Leopardus tigrinus* | 7.01 | 2.53 | 0.00 | 2.00 | 0.00 | 0.41 | 7 |
| White Tailed Deer | *Odocoileus virginianus*^+^* | 6.95 | 3.83 | 0.34 | 0.29 | 0.00 | 0.24 | 4 |
| Grey Fox | *Urocyon cinereoargenteus^+^* | 1.88 | 1.39 | 0.00 | 0.86 | 0.00 | 0.18 | 3 |
|  |  |  |  |  |  |  | **TOTAL** | **2,946** |
