# Supplementary material for: Forest cover mediates large and medium-sized mammal occurrence in a critical link of the Mesoamerican Biological Corridor
Source: PLoS One. 2021 Mar 23;16(3):e0249072. doi: 10.1371/journal.pone.0249072 (PMC7996086; doi:10.1371/journal.pone.0249072)
Supplement: S5 File — Barbilla-Destierro Biological Corridor (Corridor) and portions of Central Volcanic Cordillera (CVC) and Talamanca-Cordillera Central (TC) Jaguar Conservation Units (JCUs), surveyed with camera traps from 2013–2017. (DOCX) [file pone.0249072.s005.docx]

### S5 File: Species-level estimates for the influence of covariates on habitat use (Ψ) and detection (p) of medium and large-sized mammals. Barbilla-Destierro Biological Corridor (Corridor) and portions of Central Volcanic Cordillera (CVC) and Talamanca-Cordillera Central (TC) Jaguar Conservation Units (JCUs), surveyed with camera traps from 2013-2017.

Beta coefficients and 95% CI

Fig S1. Species-level estimates (with 95% Bayesian Credible Intervals) for the influence of human presence on habitat use (Ψ) of medium and large mammals and domestic pig (*n* = 25) in the Barbilla-Destierro Biological Corridor (Corridor) and portions of Central Volcanic Cordillera (CVC) and Talamanca-Cordillera Central (TC) Jaguar Conservation Units (JCUs), surveyed with camera traps from 2013-2017.

Beta coefficients and 95% CI

Fig S2. Species-level estimates (with 95% Bayesian Credible Intervals) for the influence of mean Enhanced Vegetation Index (EVI) on habitat use (Ψ) of medium and large mammals and domestic pig (*n* = 25) in the Barbilla-Destierro Biological Corridor (Corridor) and portions of Central Volcanic Cordillera (CVC) and Talamanca-Cordillera Central (TC) Jaguar Conservation Units (JCUs), surveyed with camera traps from 2013-2017.

Beta coefficients and 95% CI

Fig S3. Species-level estimates (with 95% Bayesian Credible Intervals) for the influence of mean distance to strictly-protected area on habitat use (Ψ) of medium and large mammals and domestic pig (*n* = 25) in the Barbilla-Destierro Biological Corridor (Corridor) and portions of Central Volcanic Cordillera (CVC) and Talamanca-Cordillera Central (TC) Jaguar Conservation Units (JCUs), surveyed with camera traps from 2013-2017.

Beta coefficients and 95% CI

Fig S4. Species-level estimates (with 95% Bayesian Credible Intervals) for the influence of mean terrain ruggedness on habitat use (Ψ) of medium and large mammals and domestic pig (*n* = 25) in the Barbilla-Destierro Biological Corridor (Corridor) and portions of Central Volcanic Cordillera (CVC) and Talamanca-Cordillera Central (TC) Jaguar Conservation Units (JCUs), surveyed with camera traps from 2013-2017.

Beta coefficients and 95% CI

Figure S5. Species-level estimates (with 95% Bayesian Credible Intervals) for the influence of effort (sum of all trap nights on each occasion) on detection (p) of medium and large mammals and domestic pig (*n* = 25) in the Barbilla-Destierro Biological Corridor (Corridor) and portions of Central Volcanic Cordillera (CVC) and Talamanca-Cordillera Central (TC) Jaguar Conservation Units (JCUs), surveyed with camera traps from 2013-2017.
