# Supplementary material for: Forest cover mediates large and medium-sized mammal occurrence in a critical link of the Mesoamerican Biological Corridor
Source: PLoS One. 2021 Mar 23;16(3):e0249072. doi: 10.1371/journal.pone.0249072 (PMC7996086; doi:10.1371/journal.pone.0249072)

Click here to access/download;Manuscript;Salom-Perez et al. Community occupancy model-Corridor_Aug10.docx

The status of medium and large-sized mammals in a critical link of the Mesoamerican Biological Corridor

Roberto Salom-Pérez[1,2,3*], Daniel Corrales-Gutiérrez[1], Daniela Araya-Gamboa[1], Deiver Espinoza-Muñoz[1], Bryan Finegan[3], Lisanne S. Petracca[4]

1 Panthera, 8 West 40th Street, 18th Floor, New York, NY 10018, USA, 2 Department of Fish and Wildlife Resources, University of Idaho, Moscow, ID 83844–1136, USA, 3 CATIE-Centro Agronómico Tropical de Investigación y Enseñanza, Turrialba, Costa Rica, 4 Department of Environmental and Forest Biology, State University of New York College of Environmental Science and Forestry, Syracuse, NY 13210, USA

* rsalom@panthera.org

Abstract

Connectivity of natural areas through biological corridors is essential for ecosystem resilience and biodiversity conservation. However, robust assessments of large-scale biodiversity are often hindered by logistical constraints and the statistical challenges of modeling data from multiple species. Herein we used a hierarchical community occupancy model in a Bayesian framework to evaluate the status of medium and large-sized mammals in a critical link of the Mesoamerican Biological Corridor (MBC): the Barbilla-Destierro Biological Corridor in Costa Rica. We used camera traps deployed from 2013-2017 to detect medium (1-15 kg) and large (>15 kg) mammal species in two protected jaguar (*Panthera onca*) core areas and the corridor linking them. Camera traps operated for 16,904 trap nights across 209 stations, covering an area of 880 km^2. We found that greater forest cover was the most important driver of medium and large-sized mammal habitat use at the community

level, with ~~lesser~~ effects ~~of~~ ~~greater~~ human presence, ~~lower~~ EVI, proximity to strictly protected areas, and ~~lower~~ terrain ruggedness. Importantly, large and medium-sized mammals were differentially affected by forest cover, with forest specialists such as jaguars and pumas (*Puma concolor*) strongly associated with forest cover, while habitat generalists such as coyotes (*Canis latrans*) and raccoons (*Procyon lotor*) ~~were~~ associated with ~~less~~ forest cover. ~~As expected,~~ the richness of medium and large-sized mammal species was lower in the corridor area ($\bar{x}$=9.78±1.84) than in the two jaguar core areas ($\bar{x}$=11.86±1.15 and $\bar{x}$=11.03±1.79). Puma (*Puma concolor*) habitat use probability followed a similar trend (Corridor: $\bar{x}$=0.30±0.21; core areas: $\bar{x}$=0.72±0.18 and $\bar{x}$=0.70±0.23). Puma and jaguar habitat use probabilities were strongly correlated with large prey species richness (jaguar, r = 0.59, $p<0.001$; puma, r = 0.72, $p<0.001$), and correlated to a lesser extent with medium prey species richness (jaguar, r = 0.36, p = 0.003; puma, r = 0.23, p = 0.064). Low estimated jaguar habitat use probability in one core area (Central Volcanic Cordillera: $\bar{x}$=0.15±0.11; compared to Corridor: $\bar{x}$=0.22±0.21; and Talamanca $\bar{x}$=0.58±0.16) suggests that this is not the jaguar stronghold previously assumed. Roads in the corridor area may be ~~having~~ a barrier ~~effect,~~ especially for large ~~prey species~~ (e.g. peccaries, deer), jaguars and pumas. Urgent actions are needed to secure connectivity of habitat for mammal populations in this critical link of the MBC, which should include restoring forest in the central-west portion of the Corridor, implementing measures to decrease the barrier effect related to the two main roads, and decreasing other potential threats (e.g. hunting), especially for large species.

Main message: Is the corridor working but not as expected? Or are more corridors needed?

Introduction

Biodiversity is essential to maintain the resilience of ecosystems and the stability of their functions [1,2]. It is also critical for supporting a range of ecosystem services, reducing the risk of spread of infectious diseases, and maintaining productivity of several agricultural systems (e.g. facilitating pollination) [1,3,4]. While forests represent one of the most biodiverse areas [5], global forest cover is being lost at a rate of 0.6% per year, largely due to conversion to agro-industrial land uses [6]. The increasing isolation of intact forest means that these natural areas may not be enough to guarantee the long-term survival of species that require large spatial extents to sustain viable populations [7,8]. Thus, connectivity between forested areas through dispersal or biological corridors in human-dominated landscapes is crucial for the conservation of wide-ranging species [9,10].

With only 0.50% of the world's land area, the Mesoamerica region is recognized as a global hotspot, holding ~7% of the world's biodiversity [11]. Because of its relatively small size and its geographic position between North and South America, this isthmus has functioned for millennia as a natural link for wildlife, becoming arguably the most critical region for habitat connectivity in the Americas. Consequently, in 1997 the governments in the region created the Mesoamerican Biological Corridor (MBC), an initiative to preserve biodiversity and connect protected and other natural areas from southern Mexico to Panama [12]. Nevertheless, forest is being lost in several parts of the corridor and some researchers have already highlighted possible areas where connectivity may be broken or close to broken [9,13–16].

Medium and large-sized mammals play an important role in ecosystem dynamics, frequently representing the bulk of wildlife biomass in a given area [17], serving as predators exerting top-down control over other vertebrates [18], or performing ecological roles as prey species [19–21], seed consumers [22–24], or seed dispersers [23,25,26]. Medium and large-

sized mammals are also among the most hunted animals, and may comprise the main source of protein for some humans [17]. Importantly, ~~the~~ large ~~body size and endothermy of large mammals creates~~ substantial energetic ~~needs~~ and therefore ~~the requirement for~~ large areas to maintain viable populations, placing many of them at higher risk of extinction and greater vulnerability to habitat alterations ~~and~~ human-related pressures [27–29].

The idea of large-sized mammals as "sentinel" species, decreasing in abundance or disappearing from areas where perturbations occur, has been a major driver of research into mammal ecology and distribution [28,30–32]. Much work has focused on single species under the "umbrella" concept, meaning that the conservation of one wide-ranging species will conserve other sympatric species [29,33–36]. However, some investigators suggest that the "umbrella" concept has not been tested appropriately [37–39], and single-species work is frequently confronted with small sample ~~numbers~~ that limit the analytical toolkit and scope of inference that can be applied [40–42].

To overcome the problems of low sample sizes and/or detection rates, researchers often turn to occupancy models. Occupancy models account for imperfect detection by incorporating detection probability [42,43], producing ~~more precise and~~ less biased estimates compared to ~~raw species counts or traditional species richness estimators~~ [44]. The extension of occupancy models to a community or multi-species framework [45–47] allows the estimation of species and community-level occupancy ~~estimates~~ by drawing species-level estimates from community-level hyperparameters [44,46–49], which can improve estimates for rare or elusive species. For these reasons, a multiple species approach to assess biodiversity, evaluate the effects of impacts or management actions, and test connectivity has proven to be a useful alternative [15,45–47,49].

~~Our goal was to~~ use a multi-species community occupancy approach [45,49] to establish baseline information ~~on the status of~~ medium and large-sized mammals in a critical wildlife corridor and ~~the~~ adjacent protected areas in Costa Rica. This area is a crucial corridor for the MBC and the Jaguar Corridor Initiative (JCI), the largest-scale carnivore conservation effort ~~existing~~ to date [9,14,50]. The JCI aims to preserve jaguar (~~*Panthera onca*; IUCN: near threatened; [51]~~) populations and range-wide habitat connectivity from Mexico to northern Argentina by identifying and securing dispersal corridors between core populations [9].

Our objectives were to: (1) determine the main environmental and human-related factors driving the presence of 24 medium (*N*=18) and large (*N*=6) native mammal species, plus the non-native domestic pig ~~that could be~~ an important prey item in the study area, (2) estimate species richness of medium and large-sized native mammal species, (3) estimate habitat use probability for jaguars and pumas (*Puma concolor*; IUCN: least concern; [52]), and (4) evaluate the relationship between jaguar and puma habitat use and prey richness. We expected that medium and large mammal species richness would be lower in the corridor ~~with respect to the~~ core areas, given that the latter ~~areas~~ have ~~greatest~~ forest cover and are classified as protected areas or indigenous territories. We also hypothesized that habitat use probability of jaguars and pumas would be related to large prey species richness given their high energetic requirements.

Materials and methods

Study area

The study area comprises the Barbilla-Destierro Biological Corridor (hereafter "the Corridor") and a portion of two Jaguar Conservation Units (JCUs): the Central Volcanic Cordillera (CVC) JCU and the Talamanca-Cordillera Central (TC) JCU (Fig 1). The JCUs are expert-defined areas that are believed to have resident jaguar populations, an adequate prey base, and high habitat quality [8,53].

Fig 1. Jaguar Conservation Units, jaguar corridors, and protected areas in Costa Rica.

The first JCU (CVC JCU) comprises a continuous block of protected areas in Costa Rica (IUCN categories II and VI; ~1,153 km^2), while the second JCU (TC JCU) is shared between Costa Rica and Panama and connects to the eastern side of the Corridor. This JCU encompasses a continuous group of protected areas (IUCN categories II and VI) and indigenous territories (~7,002 km^2) on the Costa Rican side. Primary and secondary forests represent ~75% of the JCU area, with the remaining area largely dominated by pasture [54].

The Corridor is approximately 362 km^2, of which 58% is protected (IUCN category VI; Forest Reserves and a Protected Zone) or indigenous territory with a certain level of protection. No other suitable connections for jaguars have been identified between the Central Volcanic and Talamanca JCUs, and more broadly between Nicaragua and Panama [14]. Almost all the area is privately owned, comprising small farms. About 64% of the Corridor is covered by primary and secondary forest (2015 Aster Image; AIST https://gbank.gsj.jp/madas/), with the rest of the area dominated by pasturelands for livestock (20%) and agriculture (14%). ~~The Corridor also includes~~ two two-lane paved roads that connect the towns of Turrialba and Siquirres.

Study design and data collection

We conducted camera trap surveys to assess the presence of medium (1-15 kg) and large (>15 kg) mammal species over the study area. The study area was divided in four different blocks (CVC JCU, TC JCU, and Corridor Blocks 1 and 2). We created a grid system of 63 16 km^2 cells over the entire Corridor and portions of the CVC and TC JCUs (Fig 2). Cell size represented the approximate home range size of jaguars in Central America, one of the target species in our analyses, and presumably the species with the largest home range size [55–57]. → So really the study area is not all the JCUs + corridor. Is a smaller portion centered around the corridor. Draw a rectangle around this area in figure 1.

Fig 2. Our sampling grid of 16 km^2 cells in the study area. Camera traps to survey medium and large-sized mammals were placed in the CVC JCU (August 2014 to April 2015), Block 1 (Oct 2013 - January 2014) and Block 2 (January 2014 - May 2014) of the Corridor, and the TC JCU (September 2016 to April 2017). Study area: Barbilla-Destierro Biological Corridor (Corridor) and portions of the Central Volcanic Cordillera (CVC) and Talamanca-Cordillera Central (TC) Jaguar Conservation Units (JCUs). Strictly protected areas refer to IUCN Ia & II categories.

The 16 km^2 grid cells were subdivided into four sub-cells of 4 km^2 each (Fig 3). We sampled two sub-cells per grid cell with two stations (one camera trap per station) in each sub-cell for a period of approximately three months. Selection of these sub-cells was random, but had to be adjusted when there was no forest, permits were not given, or access was difficult (e.g. presence of very steep slopes). Blocks of the study area were surveyed at different times due to limitations in the number of cameras available and logistical considerations, as well as to allow for a higher density of cameras per area. Survey periods were Oct 2013 - January 2014 (Corridor Block 1), January 2014 - May 2014 (Corridor Block 2), August 2014 to April 2015 (CVC JCU), and September 2016 to April 2017 (TC JCU).

→ sampled?

} different years and seasons

No major land use or management changes occurred during these periods, though we account for potential baseline differences in mammal habitat use across blocks via random intercepts as described later.

Fig 3. Graphic representation of a 16 km² cell, 4 km² sub-cells and the planned distribution of the camera stations in the study area. Cell: 16 km²; Sub-cells: 4 km². Black dots represent camera stations and brown lines represent human-made trails. Study area: Barbilla-Destierro Biological Corridor (Corridor) and portions of the Central Volcanic Cordillera (CVC) and Talamanca-Cordillera Central (TC) Jaguar Conservation Units (JCUs) from 2013-2017.

We placed motion sensitive camera traps (Panthera® V3, V4, V5 and V6) in forested areas, strapped to trees at approximately 0.4-0.5 m above ground. Cameras were set to function continuously and to take three shots in every event during the day and one shot during the night. One camera of each 4 km² surveyed sub-cell was placed off a trail and the other one was placed on a human-made trail (when available), in an attempt to detect species that may avoid human trails (Fig 3).

Camera location was recorded using a GPS device (Garmin®). Cameras were checked approximately every six weeks to download pictures and perform camera maintenance. Data were processed on Panthera IDS (Integrated Data Systems; version 1.13.786), where the species, date, time, and number of individuals in each photograph were recorded. For the occupancy-based analyses, we collated data from all stations in each grid cell to make detection histories per week ("1" = detected, "0" = not detected, "NA" = inactive camera) for each of the 24 medium (*N*=18 species) and large (*N*=6) native mammal species, plus domestic pig (Total *N*=25) (Table 1). We included domestic pig as they were frequently recorded and could represent an important prey item in the study area for jaguars and pumas, especially in

indigenous areas where they roam freely in the forest (R. Salom-Perez, *personal observation*).

Table 1. Medium and large-sized mammal species included in the global analysis (N=25). Those included in the habitat use analysis for jaguars and pumas are indicated by a check mark. Barbilla-Destierro Biological Corridor and portions of the Central Volcanic Cordillera (CVC) and Talamanca-Cordillera Central (TC) Jaguar Conservation Units (JCUs), surveyed with camera traps from 2013-2017.

Medium-sized species	Common name	Included as prey for jaguars in the analysis	Included as prey for puma in the analysis
Cabassous centralis	Northern naked-tailed armadillo		
Canis latrans	Coyote		
Conepatus semistriatus	Skunk	✓	✓
Cuniculus paca	Paca	✓	✓
Dasyprocta punctata	Agouti	✓	✓
Dasypus novemcinctus	Nine-banded armadillo	✓	✓
Didelphis marsupialis	Opossum	✓	✓
Eira barbara	Tayra		✓
Galictis vittata	Grison	✓	
Nasua narica	Coati	✓	✓
Leopardus pardalis	Ocelot		
Leopardus wiedii	Margay		
Leopardus tigrinus	Oncilla		
Procyon lotor	Raccoon	✓	✓
Puma yagouaroundi	Jaguarundi		
Sylvilagus brasiliensis	Rabbit	✓	✓
Tamandua mexicana	Tamandua	✓	✓
Urocyon cinereoargenteus	Gray fox		✓
**Large-sized species**	**Common name**	**Included as prey for jaguars in the analysis**	**Included as prey for puma in the analysis**
Mazama temama	Red-brocket deer	✓	✓
Odocoileus virginianus	White-tailed deer	✓	✓
Panthera onca	Jaguar		
Pecari tajacu	Collared peccary	✓	✓
Puma concolor	Puma		
Sus scrofa	Domestic pig	✓	✓
Tapirus bairdii	Tapir		

We selected site covariates *a priori* as thought to be the main drivers of habitat use of medium and large sized-mammals in the study area [9,14,15,53,58,59] (Table S1 in S1 File). Site covariates expected to have a positive relationship with the habitat use of medium and

large mammals were enhanced vegetation index (EVI) [60], forest cover [59], distance from any settlement, and distance from major settlements (main settlements in Provinces, Counties and Districts excluding National Geographic Institute (IGN) exclusive areas) [59]. Site covariates expected to have a negative relationship with the habitat use of medium and large mammals were elevation [61], terrain ruggedness [62], distance to strictly protected areas (IUCN Ia & II categories) [59], distance to JCUs (Panthera unpub. data) and human presence (calculated as the number of human detections per 1,000 trap nights per stations; S1 File). All covariates were centered and standardized prior to inclusion in the models such that the magnitude of regression coefficients of all covariates could be compared within and among models [63]. To calculate effort, a covariate on detection, we calculated and standardized the sum of all trap nights on each occasion for every grid cell.

Multi-level community occupancy model

Given that our survey occurred over a long period of time (~3.5 years), the closure assumption (i.e., there are no changes in the occupancy status of grid cells during the survey period [43]) is likely violated. Thus, in this investigation occupancy probability (Ψ) can be interpreted as probability of habitat use [64], and we assume that any changes in the occupancy status of grid cells over our survey period were random.

We used a multi-level (or hierarchical) community occupancy model in a Bayesian framework to calculate jaguar and puma habitat use probability and obtain medium and large-sized mammal species richness [45–47,49]. Unlike traditional occupancy models, the Bayesian framework can account for unobserved heterogeneity across species, space or time through random effects [14]. This type of heterogeneity could be expected in the current

investigation given the relatively large spatial extent and the fact that the blocks were surveyed in different time periods.

Our final hierarchical occupancy model included forest cover, human presence, EVI, distance to strictly-protected area, and terrain ruggedness on habitat use, and effort on detection (Tables S1 and S2 in S1 File). Habitat use probability for each species at each site was estimated as:

$$\text{Logit}\,(\Psi_{i,j}) = \xi_{il} + \alpha_i D_j \qquad (1)$$

where $\xi_{il} \sim \text{Normal}(\mu_\xi, \tau_\xi)$ is the random intercept for each species i at each block l (CVC JCU, Corridor Blocks 1 and 2, and TC JCU), μ_ξ is the community-level or hyperparameter mean for the intercept on habitat use, and τ_ξ is its precision. The random intercept was used to account for potential spatial and temporal heterogeneity in habitat use due to differences by species and survey block [46,47,49]. In addition, α_i are estimated beta coefficients on habitat use for species i, where $\alpha_i \sim \text{Normal}(\mu\alpha, \tau\alpha)$, $\mu\alpha$ is the community-level or hyperparameter mean for each beta coefficient, and $\tau\alpha$ is its precision. D_j are the standardized values for each covariate at grid cell j.

Detection probability for each species i at each site j and in each week k, was estimated as

$$\text{Logit}\,(p_{i,j,k}) = v_{il} + \beta_i effort_{j,k} \qquad (2)$$

where $v_{il} \sim \text{Normal}(\mu_v, \tau_v)$ is the random intercept for each species i at each block l, μ_v is the community-level mean for the intercept on detection, and τ_v is its precision. In addition, β_i is the estimated beta coefficient for effort for species i, where $\beta_i \sim \text{Normal}(\mu_\beta, \tau_\beta)$, μ_β is the community-level mean for effort, τ_β is its precision, and $effort(j,k)$ are the standardized values for effort at grid cell j on occasion k.

We defined true occurrence $z_{(i,j)}$ as a binary variable in which $z_{(i,j)} = 1$ if species i occurred in grid cell j and $= 0$ otherwise. We modeled occurrence from a Bernoulli random variable, where $z_{(i,j)} \sim \text{Bern}(\Psi_{i,j})$, where $\Psi_{i,j}$ is the probability that species i occurs at grid cell j. To account for imperfect detection, we modeled observed data $y(i,j,k)$ as $\text{Bern}(p_{i,j,k} * z(i,j))$, where $p_{i,j,k}$ is the detection probability of species i in grid cell j in the survey occasion (week) k. The model accounted for the effect of species abundance on detection probabilities via species correlation parameter rho (ρ), a correlation between habitat use and detection probability [45].

We fit the Bayesian models in R 3.5.1 (R Core Team 2018) using package jagsUI (59), specifying three MCMC chains of 30,000 iterations, a burn-in of 5,000, and a thinning rate of three.

We estimated jaguar and puma habitat use probability as a function of species richness from the community model. Species richness per grid cell was calculated as a derived parameter from the summation of $z(i,j)$ values. We calculated species richness for (1) all medium (N=18) and large-sized (N=6) native mammal species, including jaguar and puma and excluding the domestic pig; (2) jaguar and puma large prey species, including domestic pig (N=4); (3) jaguar medium prey species (N=10); and (4) puma medium prey species (N=11 species). In order to select the medium and large prey species for jaguars and pumas, we conducted a literature review of publications on jaguar and puma diet and predation reports from Mexico to Panama (Table S4 in S3 File).

Lastly, we estimated the correlation of jaguar and puma habitat use probabilities with (1) medium prey richness and (2) large prey species richness, both of which were derived parameters from the prey community occupancy model, in order to evaluate if areas with

higher probability of jaguar or puma habitat use were more strongly associated with large or medium prey richness.

Results

Camera traps operated for 16,904 total trap nights across 209 stations. Fifty-five out of 63 total cells were surveyed, covering 87.30% of the study area (880 km^2). We registered 2,946 independent records of medium (*N*=19) and large (*N*=6) mammal species, including domestic pig (Table S5 in S4 File).

what was considered an independent record?

The five most widespread species estimated by the Bayesian occupancy model were nine-banded armadillo (occurring in an estimated 89.32% of the area), coati (86.87%), ocelot (85.94%), tayra (82.35%) and jaguarundi (70.53%) (Table S5 in S4 File).

The top drivers of habitat use of medium and large-sized mammals were greater forest cover (α=0.34; 95% Credible Interval (CRI) -0.21, 0.90), greater human presence (α=0.10; 95% CRI -0.12, 0.31), lower EVI (α=-0.08; 95% CRI -0.57, 0.39), closer distance to strictly-protected area (α=-0.08; 95% CRI -0.47, 0.30), and lower terrain ruggedness (α=-0.02; 95% CRI -0.37, 0.32) (Fig 4, Table 2). Detection probability was positively associated with effort, the sum of all trap nights on each occasion (*β*=0.39; 95% CRI 0.30, 0.49). There was a positive correlation between habitat use and detection (*ρ*=0.26; 95% CI=-0.04 to 0.91). → not strong

overlap zero. No effect.

Fig 4. Community-level hyperparameter estimates (with 95% Bayesian Credible Intervals) for habitat use (*Ψ*) of medium and large wild mammals and domestic pig (*N*=25). Estimates with a negative influence are shown in white, and the ones with a positive influence are shown in black. Barbilla-Destierro Biological Corridor (Corridor) and portions of Central Volcanic Cordillera (CVC) and Talamanca-Cordillera Central (TC) Jaguar Conservation Units (JCUs), surveyed with camera traps from 2013-2017.

Table 2. Community-level summary of hyperparameters for the covariates on detection and habitat use in the top model driving occurrence of medium and large wild mammals and domestic pig (n = 25). We present the posterior means with standard deviation and 95% credible intervals, and an indicator of convergence ($\hat{R}$), for which values <1.1 indicate convergence. Barbilla-Destierro Biological Corridor and portions of the Central Volcanic Cordillera (CVC) and Talamanca-Cordillera Central (TC) Jaguar Conservation Units (JCUs), surveyed with camera traps from 2013-2017.

either this or Figure 4. Do not need both.

Model parameter	Covariates	Beta (SD)	95% credible interval	50% credible interval	$\hat{R}$
Habitat use (Ψ)	Percent forest version 2	0.34 (0.28)	-0.21, 0.91	0.16, 0.52	1.000
	Human presence (human detections per 1,000 trap nights)	0.10 (0.11)	-0.12, 0.31	0.03, 0.17	1.002
	Mean Enhanced Vegetation Index	-0.08 (0.24)	-0.57, 0.39	-0.23, 0.08	1.000
	Mean distance to strictly-protected area	-0.08 (0.19)	-0.47, 0.30	-0.20, 0.04	1.001
	Mean Ruggedness	-0.02 (0.18)	-0.37, 0.32	-0.13, 0.10	1.000
Detection (p)	Effort (sum of all trap nights on each occasion)	0.39 (0.05)	0.30, 0.49	0.36, 0.42	1.000

All variables informing habitat use had a 95% CI overlapping zero, suggesting variability of covariate effects among species (Figs 4 and 5; Figs S1-4 in S5 File) [45]. Percent forest had the greatest influence on species richness, with 89.26% of its posterior distribution above

This is inaccurate. There is large variability between species

0 and an effect size more than three times that of any other variable (Fig 4). At the species level, this relationship was strongest (i.e., 95% CI did not overlap 0) in the positive direction for collared peccary, jaguar, domestic pig, paca, puma, agouti and ocelot, while there was a strong negative effect for coyote, nine-banded armadillo and raccoon (Fig 5).

Fig 5. Community-level hyperparameter estimates (with 95% Bayesian Credible Intervals) for the influence of forest cover on habitat use (Ψ) of medium and large mammals and domestic pig (*N*=25 species). Barbilla-Destierro Biological Corridor (Corridor) and portions of Central Volcanic Cordillera (CVC) and Talamanca-Cordillera Central (TC) Jaguar Conservation Units (JCUs), surveyed with camera traps from 2013-2017.

Medium and large mammal species richness estimates per grid cell ranged from 6.02 to 14.54 (95% CI 5-16; $\bar{x}$ = 10.60±1.90) of 24 total species (domestic pig not included), with the TC JCU having the highest species richness estimate overall ($\bar{x}$ = 11.86±1.15), followed by the CVC JCU ($\bar{x}$ = 11.03±1.79), and Corridor ($\bar{x}$ = 9.78±1.84) (Fig 6a).

Fig 6. Richness of native (a) medium and large-sized mammals, (b) large prey, (c) medium jaguar prey, and (d) medium puma prey. The two main roads are shown in dotted lines; and the strictly protected areas are shown as striped polygons. Barbilla-Destierro Biological Corridor (Corridor, white polygon) and portions of Central Volcanic Cordillera (CVC) and Talamanca-Cordillera Central (TC) Jaguar Conservation Units (JCUs) (black outline), surveyed with camera traps from 2013-2017.

Jaguar and puma large prey species richness was distinctly higher in the TC JCU ($\bar{x}$ = 1.53±0.52), followed by CVC JCU ($\bar{x}$ = 1.25±0.63) and lowest in the Corridor area ($\bar{x}$ = 0.59±0.59), especially in the west-central section (Fig 6b). On the other hand, jaguar and puma medium prey species richness was higher in the eastern side of the Corridor (Block 2; jaguar medium prey (jmp): $\bar{x}$ = 6.14±1.02; puma medium prey (pmp): $\bar{x}$ = 6.87±0.94) in

comparison to the rest of the Corridor (Block 1; jmp: $\bar{x}$ = 4.71±1.13; pmp: $\bar{x}$ = 5.41±1.02), the CVC JCU (jmp: $\bar{x}$ = 4.88±1.16; pmp: $\bar{x}$ = 5.23±1.01) and the TC JCU (jmp: $\bar{x}$ = 5.78±0.79; pmp: $\bar{x}$ = 6.50±0.7.0) (Fig 6c,d).

Jaguars were less widespread in the study area (estimated to occur in 29.74% of the study area) compared to pumas (49.68%) (Fig 7a,b). Puma habitat use probability was lower in the Corridor ($\bar{x}$ = 0.30±0.21) in comparison to the core areas (TC JCU: $\bar{x}$ =0.72±0.18 and CVC JCU: $\bar{x}$ = 0.70±0.23). On the other hand, jaguar habitat use was higher in the TC JCU ($\bar{x}$ = 0.58±0.16), followed by the Corridor ($\bar{x}$ =0.22±0.21) and the CVC JCU ($\bar{x}$ =0.15±0.11). Jaguar and puma estimates of habitat use were strongly correlated with large prey species richness (jaguar, r = 0.59, p<0.001; puma, r = 0.72, p<0.001), and correlated to a lesser extent with medium prey species richness (jaguar, r = 0.36, p = 0.003; puma, r = 0.23, p = 0.064).

Fig 7. Habitat use probability for (a) jaguar and (b) puma. The two main roads are shown in dotted lines; and the strictly protected areas are shown as striped polygons. Barbilla-Destierro Biological Corridor (Corridor, white polygon) and portions of Central Volcanic Cordillera (CVC) and Talamanca-Cordillera Central (TC) Jaguar Conservation Units (JCUs) (black outline), surveyed with camera traps from 2013-2017.

Discussion

To our knowledge this is the most intensive camera trap study ~~on a continuous area in Costa Rica [65], and the first~~ to take place in this critical link between two jaguar core areas. Herein we found that forest cover was the primary driver ~~of~~ medium and large-sized mammal habitat use at the community level, with evidence of widely differing relationships with forest cover at the level of individual species. In addition, the status of the Corridor with respect to medium and large-sized mammals is less than optimal as illustrated by the low species

yes, but it is really good!
Is it statistically significant!

richness values ($\bar{x}$ = 9.78±1.84 spp. compared to 11.50±1.52 in core areas) and relatively low number of records, especially of large-sized species. Additional research on the jaguar core area in the west is necessary to evaluate the viability of this critical link of the MBC and the JCI for jaguars, as jaguar records were primarily on the east side of the study area. This baseline information will be of paramount value to measure the outcome of conservation initiatives in the years to come, especially related to the recently-constructed hydroelectric project in the middle of the corridor and related mitigation actions (R.Salom-Perez et al. *in prep.*).

→ got to be careful about assigning causation

Given our finding that forest cover was the most important driver of medium and large-sized mammal habitat use, there were lesser effects of greater human presence, lower EVI, proximity to strictly protected areas, and lower terrain ruggedness. Forest cover had an effect that was at least three times higher than any other covariate, and seemed to be especially important for species including the collared peccary, jaguar, domestic pig, puma, agouti and ocelot. These species, with exception of the domestic pig, are known to depend on, or at least be associated with, vegetation cover [51,52,66–68]. Almost all domestic pigs we detected were in the eastern side of the corridor, specifically in or near indigenous territories with high forest cover. These animals belong to the indigenous people and roam freely in the forest. On the other hand, coyote, nine-banded armadillo and raccoon seem to avoid areas with high forest cover in the study area. This was not surprising, as these are adaptable species that can be found in open and/or disturbed areas [66,69–71].

distribution was wider

Contrary to our predictions, human presence and EVI were positively and negatively associated with overall species habitat use, respectively, though both effects were weak (i.e. the 95% CIs highly overlapped zero). While human presence is often used as a proxy for disturbance and has found to be negatively associated with habitat use at the community level

its binary; it overlaps or not.

[47], in our case the presence of humans may have a different interpretation. For example, people in our study area (e.g. tourists, hunters, indigenous people) may be actively looking for these animals and selectively walking on the same trails or in the same areas where they occur, leading to a positive association with mammal habitat use. This potentially important result requires additional study.

You located camera traps in trails. Isn't this expected?

As we anticipated, medium and large-sized native mammal species richness was lower in the Corridor area than in the two JCUs. The lowest values for richness for these species, and for jaguar and puma habitat use probabilities were in the western half of the corridor, with low jaguar habitat use extending inside the core area to the west. Most of these low-probability grid cells in the Corridor are situated between the two main roads. Although the proximity to road covariate did not make our global habitat use model, this and other types of infrastructure are known to have a negative effect on the presence of certain mammal species [31,72,73]. It seems that large prey species, jaguars and pumas may be able to get close to the roads, but will seldom cross and establish territory in the area between them. Additionally, these areas have less forest cover and higher settlement density, factors highly related to the presence of roads. We recommend further investigation into the barrier effect of these roads (and not just evaluate road proximity or density), such as the use of telemetry and non-invasive genetic tools to investigate movement and gene flow [13,72,74].

The highest habitat use probability for jaguars was located inside or near a strictly protected area, Barbilla National Park (IUCN category II) in the eastern core area (TC). On the other hand, it was surprising to see that jaguar habitat use probability in the western core area (CVC) was very low, in spite of its apparent suitability for this species [50,59]. However, jaguar presence is likely not supported by other characteristics of the area, as most of the surveyed area in this JCU is not strictly protected (IUCN category VI), the terrain is very

rugged and some areas have high elevation (2,000-3,300 m.a.s.l.) [51,75]. In contrast, pumas have high habitat use probability in both JCUs. Pumas are known to occur more frequently in higher elevations than jaguars, and could be benefitting from the apparent absence of a direct competitor in the CVC JCU [52,75,76]. A recent investigation further west into the CVC JCU found no sign of jaguar, adding extra support to the hypothesis that this area is not the jaguar stronghold previously assumed (Velado et al. unpub. data). It remains to be established whether low jaguar presence in this JCU is explained by the site conditions mentioned above or if it is the result of more historical pressures (e.g. hunting, isolation).

history of hunting in this area?

Regarding the role of large carnivores as "umbrella" species [77,78], we found that puma and jaguar habitat use probabilities were strongly correlated with large prey species richness and, to a lesser degree, with medium prey species. These results indicate that conservation actions directed to increase presence of these two carnivores should also improve the habitat conditions for prey species, and that jaguars and pumas may serve as umbrella species for most large and medium-sized mammals in the area. However, we recommend additional studies that would include other taxa to test the umbrella value of these large carnivores over a broader number of species. When possible, the use of a group of species with different habitat requirements to plan a conservation strategy and monitoring programs should be favored [45,46,49,79,80]. However, when resources are scarce, as it frequently occurs in the conservation world, the use of one or few verified umbrella species may be of great value [29,35,36,81].

Conclusions

The multi-species community occupancy approach using camera trapping data presented here generates an important evidence base for the management and monitoring of a mammal assemblage. The hierarchical community approach allows for the estimation of species and community-level effects, and allows for the incorporation of data from rare species, as is the case with several mammal species. The model is versatile, accounting for heterogeneity in the sampling process (e.g. data taken over several sites or different years) through the incorporation of random effects, and thus can be easily adapted to different survey conditions.

This is all about the model.

Our results highlight the importance of generating on-the-ground information on the status of multiple species within core and corridor areas, as well as using a hierarchical modeling framework as the basis for management recommendations at the community and individual species levels. Further research should be made on the effect of roads on jaguars, pumas and large prey species, as roads may be an important barrier for regular movements and dispersal between core areas. Urgent actions are needed to secure connectivity of mammal populations in our study area and within the greater Mesoamerican Biological Corridor, and should focus on restoring areas in the central-west portion of the Corridor, notably between the two main roads, and increasing habitat quality for prey species.

Acknowledgments

We thank Drs. Lisette Waits, Daniel Thornton, Fernando Casanoves, Ryan Long, Hugh Robinson, Nathaniel Robinson, Howard Quigley, and MSc. Carlomagno Soto for their valuable support and comments. We would like to thank the numerous technicians, researchers and volunteers that collaborated on the field work and data processing. We would

Fig_1

Click here to access/download;Figure;Fig_1.tif

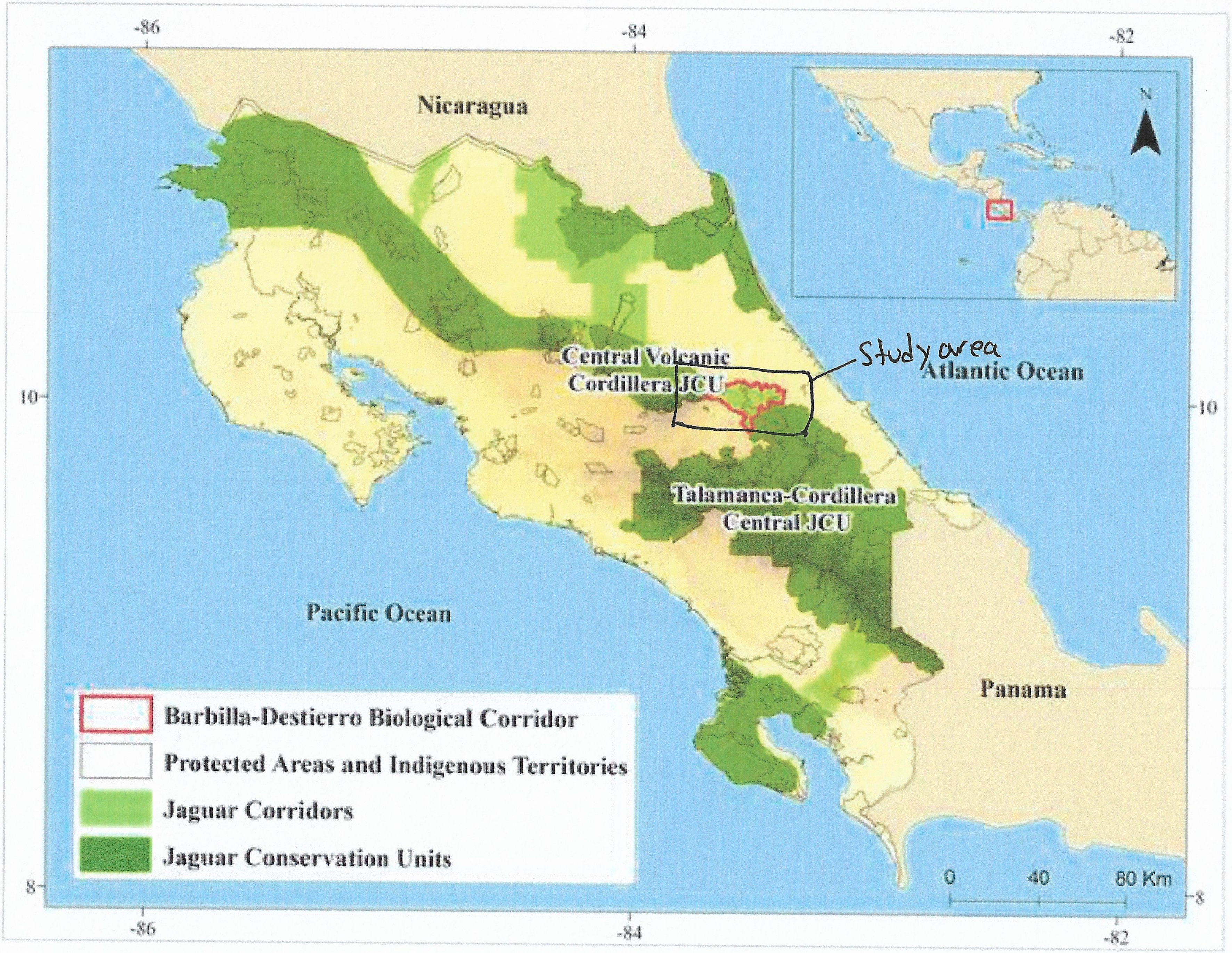

Supplement: Attachment — Submitted filename: Review of PONE-D-20-25172.pdf [file pone.0249072.s006.pdf]
