# Supplementary material for: Forest cover mediates large and medium-sized mammal occurrence in a critical link of the Mesoamerican Biological Corridor
Source: PLoS One. 2021 Mar 23;16(3):e0249072. doi: 10.1371/journal.pone.0249072 (PMC7996086; doi:10.1371/journal.pone.0249072)
Supplement: Attachment — Submitted filename: PlosONE Response to reviewers-D-20-25172.docx [file pone.0249072.s007.docx]

**Original Manuscript ID:** PONE-D-20-25172

**Original Article Title: “**The status of medium and large-sized mammals in a critical link of the Mesoamerican Biological Corridor”

**To:** PLOS ONE Editorial Board

**Re:** Response to reviewers

To the Editorial Board at PLOS ONE,

Thank you for the effort you have spent in handling our manuscript, and for giving us the chance to respond to these two thoughtful and thorough reviews. You will note our responses in blue below.

We are sending 1) our response to reviewers’ comments (below), 2) a marked-up copy of our manuscript with tracked changes, and 3) an unmarked version of our revised paper without tracked changes.

Best regards,

___________________

Roberto Salom-Pérez

Costa Rica and Mesoamerica Director

Panthera

T. (506) 2234-8079/Cel. (506) 8538-4178

E. rsalom@panthera.org

1. Please ensure that your manuscript meets PLOS ONE's style requirements, including those for file naming. The PLOS ONE style templates can be found at

https://journals.plos.org/plosone/s/file?id=wjVg/PLOSOne_formatting_sample_main_body.pdf and

https://journals.plos.org/plosone/s/file?id=ba62/PLOSOne_formatting_sample_title_authors_affiliations.pdf

**Authors´Comment:** We have carefully revised our manuscript completely according to the PLOS ONE style requirements. We made several changes to the first page (title, authors, affiliations). We´ve also included some changes in the format in the main body.

2. In your Methods section, please provide additional information regarding the permits you obtained for the work. Please ensure you have included the full name of the authority that approved the field sites access and, if no permits were required, a brief statement explaining why.

**Authors´Comment:** We´ve included the information on the entity providing the permits in the Methods section.

3. We note that Figures 1, 2, 6, 7 in your submission contain map images which may be copyrighted. All PLOS content is published under the Creative Commons Attribution License (CC BY 4.0), which means that the manuscript, images, and Supporting Information files will be freely available online, and any third party is permitted to access, download, copy, distribute, and use these materials in any way, even commercially, with proper attribution. For these reasons, we cannot publish previously copyrighted maps or satellite images created using proprietary data, such as Google software (Google Maps, Street View, and Earth). For more information, see our copyright guidelines: http://journals.plos.org/plosone/s/licenses-and-copyright.

**Authors´Comment:** Figures 1, 2, 6 and 7 are the original work of the authors using original information, public layers from Panthera (the organization of the lead author) and the Costa Rican public Atlas (Ortiz-Malavasi E. Atlas digital de Costa Rica 2008. Cartago, Costa Rica; 2009). We are providing a permission letters from Panthera and TEC/Ortiz-Malavasi.

We have also ensured that our full data are now freely available on the senior author’s GitHub page (please see <https://github.com/lisannepetracca/Salom_Perez_et_al_2021_PLOSOne>).

**Review Comments to the Author**

Reviewer #1: I found the present manuscript well written and presenting very valuable information that can contribute to the design and implementation of biological corridors.

Some observations I have are:

Authors are hypothesizing that presence of puma and jaguar is associated with large prey species richness. However, in the Introduction I did not observe a rationale that supports that hypothesis. Arguably, prey biomass is more important than richness to determine predator presence. For example, in Los Llanos of Venezuela, 70% of jaguar diet is made up of 4 species (2 peccaries, capybara and giant ant eater) (Polisar et al 2003); a similar case is observed by Aranda (1994) in Mexico and other studies. It will be helpful to expand the introduction to address this hypothesis.

**Authors’ comment:** This is a great point by the reviewer, and we can certainly see the argument that prey biomass is a core component determining presence of predators. However, it has also been found that the richness of large-bodied prey can be a major driver of predator presence at large scales (please see Petracca et al. 2018 in *Journal of Applied Ecology* for an example including all jaguar corridors within Central America). It was the strong effect of this covariate in the Petracca et al. 2018 paper that led us to include this covariate in our manuscript - we have added material in our Introduction (L. 105-109) to help support our hypothesis that prey richness can be a viable driver of predator occupancy.

Results of most covariates used to describe habitat use by predators and prey are non-conclusive. For example, in the case of human presence, distance to protected area and terrain ruggedness, 95% CRI overlap zero for all species. Only forest cover and EVI present conclusive results for some species (i.e., CRI do not overlap zero). As the first objective of this work is to “determine the main environmental and human-related factors driving the presence of 24 medium (N=18) and large (N=6) native mammal species…”, I suggest authors should test the effect of other predictors that might be of greater importance to determine species presence. For instance, authors mention the possible effect of roads on jaguar and puma. I think authors should include a measure of roads (e.g., distance to roads or density of roads) in their models, and remove covariates that seem to be not contributing to describe habitat use or species presence. Current work that evaluates the effect of roads on jaguar abundance can support that analysis, for example, see Espinosa et al. 2018: https://doi.org/10.1371/journal.pone.0189740.

**Authors’ comment:** Thank you for this valuable input. Given the feedback from both reviewers, it is obvious that we should have been much more clear about how our final Bayesian multi-species occupancy model was selected. We direct the reviewer to L. 209-221, where we have described the process of how we started with 11 covariates and used a maximum likelihood model selection approach to determine our final model. This protocol followed the statistical procedure of Petracca et al. 2018, mentioned above. Thus, we did indeed test for the explanatory value of other covariates, including distance to roads, but found them to be inconsequential compared to the final selected covariates. Please see Supplementary Material S1 & S2 for detailed information on our model selection process.

As to the observation that the effects of some covariates are inconclusive at the community level (and we expand upon this further in a response to Reviewer 2): this overlap of 95% CRIs with zero is common at this level due to (1) general uncertainty, given that certain species were detected very infrequently due to low detection probabilities, and (2) there is species-specific variation in parameter estimates. Examples of published papers with 95% community-level hyperparameters overlapping zero are Petracca et al. (2019) in *Animal Conservation* and Rich et al. (2016) in *Journal of Applied Ecology*. This was our motivation for providing 50% CRI estimates as well -- to provide a bit more nuance as to where the 25th to 75th percentile posterior densities were falling. We would also hesitate to remove certain covariates just because they are inconclusive at the community level, as their inclusion may prove important at the level of species-specific estimates.

I found the results of forest cover as a predictor of species presence very interesting; with a positive relationship with animals sensitive for habitat degradation, and a negative relationship with opportunistic species that “flourish” in an anthopogenized landscape. I think authors could put more emphasis on this finding and expand their discussion on this topic. Also, based in that finding I think authors should explore different models (i.e., different sets of covariates) for different species guilds or groups. For example, for small and less hunted species, habitat covariates, such as forest cover, proximity to water, terrain ruggedness among others might be more important than human-related covariates. However, for highly hunted species, such as peccaries, or species undergoing retaliatory killing, such as jaguar and puma, human-related covariates might be more important in determining their presence (e.g., human density, distance to road, distance to towns, etc.).

**Authors’ comment:** We completely agree that we should have placed more emphasis on our finding that forest cover had different impacts depending on species’ sensitivity to habitat degradation. Please see L. 395-406 for how we have expanded upon this finding in the Discussion.

As to your second point about delineating species guilds or groups, the big advantage of using a multi-species occupancy model is that the integration of data for all species allows for a “drawing of strength” such that species-specific parameters are estimated with greater precision (Dorazio & Royle 2005; Zipkin et al., 2009). This is particularly true for those species that were rarely photographed due to low detection probabilities. Thus, if we were to (post facto, which is likely problematic) divide the species into guilds and employ different covariates within each submodel -- that indeed goes against the very reason why we employed a multi-species model in the first place, and would almost certainly lead to less precision in our estimates and an inability to compare effect sizes across species.

Lastly, the parameterization of our model still allows for the estimation of species-specific effects on occupancy. If covariates are not important for a given species, the species-specific estimates will simply have a posterior density around zero; if the covariate is indeed important, the estimates will have posterior densities that do not overlap zero (please see Figure 5 for an example of how much species-specific estimates ranged for forest cover). Thus, our model, as we have parameterized it, should not affect our ability to determine what covariates are important (or not) at the species level.

For the reader, it would be helpful to organize the manuscript in different subsections, for example, in methodology: Study area, wildlife surveys, Statistical analyses, etc. Also, Results and Discussion could be organized under the two hypothesis authors are testing: 1) species richness is higher in better conserved areas, 2) habitat use of large predators is related with large prey species richness.

**Authors’ comment:** Great suggestion. We have improved our manuscript organization adding subsections in the Results section.

Minor comments:

Line 124-125: “. This JCU…” not clear if it is the first or the second, clarify

**Authors’ comment:** We´ve changed the wording to specify the JCU we are referring to.

Table 2. I think there is a mistake in a covariate name “Percent forest version 2”; correct in final version. Why is a 50% CRI being reported? I think there is no need for that.

**Authors’ comment:** We´ve changed the name of the covariate.

A 50% CRI is often reported to provide information on the magnitude and directionality of what is essentially half of the parameter estimate’s posterior density. Please see Petracca et al. (2019) in *Animal Conservation* for an example of 50% CRIs in the published literature. It is often helpful to use when the 95% CRI of a hyperparameter overlaps zero, but the 25th through 75th percentiles of the posterior density do not. It provides more nuance when it is often assumed that, just because a 95% CRI overlaps zero, there is no effect or directionality of that effect.

Line 356: Consider not citing work “in preparation”; not a real reference.

**Authors’ comment:** We´ve deleted this reference.

Lines 385-386: This is a speculation that needs some justification; whether jaguar or pumas establish between roads depends on how far apart roads are, what is between roads and along them.

**Authors’ comment:** We agree, we´ve changed the wording to better explain our point.

Reviewer #2: The paper is an assessment of the mammal fauna in this area (which happens to have a corridor), coupled with a more in-depth analysis of the underlying drivers affecting the occupancy of jaguars and pumas in the region. Being in a corridor (or not) does not seem to be a contrast to be estimated in the model although the authors do compare some of the results between the three sub-study areas (one in a corridor and two not). As such the paper seems unfocused covering several questions with little rigor and no insights into the corridor issue itself.

**Authors’ comment:** Thanks for this input, and for your thoughtful review in general. We did indeed think about employing “corridor” as a categorical covariate in our modeling framework. However, we decided against this, hypothesizing that instead a continuous representation of these ideas (i.e., distance from strictly protected area and distance from Jaguar Conservation Unit at the grid level) would be more appropriate. Our motivation was our earlier work (Petracca et al. (2018)), which found “distance to strictly protected areas” to be an important covariate explaining jaguar habitat use within corridors in Central America.

It is also important to consider that not all corridor areas are equivalent, with corridor units closer to JCU or protected area boundaries less likely to be degraded. It is also straightforward to derive estimates of richness by study area (corridor or not) post facto within a Bayesian framework, so we figured we would be able to investigate the corridor vs. non-corridor aspect without making “corridor” a categorical variable explicitly. This information may prove to be valuable to monitor species richness changes in time both in the corridor and in the adjacent areas (strictly protected areas or JCUs) that have different management types.

I have issues with the design of the study, in particular whether camera trap deployments were combined for a given cell or considered as independent measurement units within the cell. Also, camera traps were deliberately placed in trails, which can bias the measurement towards some species and increase the number of people captured. This is not ideal given that the assessment has a large geographical breadth and these placement decisions can affect the magnitude and precision of covariates at regional scales. The authors need to justify this design more clearly, explain how observations were aggregated for analysis, and explain what they mean by “independent observations”.

**Authors’ comment:** Data were combined from camera traps within each cell, as described in Lines 197-200. Additionally, about half of the cameras were placed on trails, but the other half was placed off trails as there are species that favor walking on trails but others avoid them, as you rightfully indicate. This was stated in Lines 190-192 and Figure 3.

I was happy to see the use of modern statistical multi-species models being used in the analysis of these data and correcting for detection probability. However it was unclear how the authors arrived at their final model and how covariates were selected. I was disappointed that corridor/non-corridor was not a covariate in itself. The interpretation of some of the effects is incorrect (e.g. Fig 4) describing effects as positive or negative, when in reality they overlap with 0. The description of the statistical significance of some of the correlation coefficients is incorrect as well. When describing coefficients, effects or expected values the authors switch between mean+-confidence intervals and mean +- ? (Unclear wether it is standard error or standard deviation). For example when comparing the species richness between the three study areas (lines 312) it is unclear which parameter is used to describe uncertainty. If these are standard errors, there might not be any real difference between richness. If it is standard deviation, there might or might not be, the reader cannot assess it.

**Authors’ comment:** Thanks for this input, and for the praise that we are indeed employing a more modern statistical method (much appreciated!).

In response to your comments, we acknowledge that we were not explicit enough in describing how we arrived at our final model (which was actually a maximum likelihood model selection strategy drawn from Petracca et al. (2018) in *Journal of Applied Ecology*). Reviewer 1 stated this as well. Hence, we have added material to the ms (L. 209-221) and have directed readers to the Supplementary Material for a full description of our approach.

In addition, when one sees a 95% Bayesian credible interval that overlaps zero, a common reaction is to assume that the covariate has “zero” effect and that the effect is “non-significant.” The use of Bayesian statistics is a bit more nuanced, such that the overlap of a 95% BCI with zero does not mean there is no effect, but rather that there is imprecision in that estimate. We can indeed say that the parameter point estimate is positive or negative, but that there is imprecision. Don’t get me wrong, if a posterior density is centered directly on zero (please see our terrain ruggedness output for an example), we can be fairly confident that there is little to no effect. However, the 50% CRIs for two hyperparameters (percent forest cover and human presence) were above zero (and 90% of the posterior density for percent forest was above 0), suggesting that there is likely a directionality of that effect despite the imprecision (see Table 2; please also see Rich et a. 2016 in *Journal of Applied Ecology* for a highly-cited example of community-level hyperparameters having 95% BCIs that overlap zero). In general, having hyperparameters of low precision is common, especially if you are working with species with low detection probabilities and/or there is great variation in species-specific responses - luckily, we were able to see more precise estimates at the species level, using percent forest cover as an example in Figure 5.

As to our precision, for our Bayesian parameter estimates (i.e. the covariate betas) we provide the 95% CRI and 50% CRIs, which represent common summaries of posterior densities. You are correct that we failed to provide the unit of precision for our species richness estimates, which were derived parameters within the Bayesian model (rather than explicitly estimated parameters as the covariate parameters were). The unit is indeed SD here and we note that in the estimates provided.

Both in the results and the discussion the authors seem to be more focused on the signs of the effects, than in their magnitude. Many of the effects discussed are not surprising (e.g. more forest more species). But there could be so much more here! In my opinion, the results —as presented— represent a lost opportunity since they might have important implications to better understand the effectiveness of corridors for mammals. As an example, if species richness within the corridor is not-significantly different compared to the more intact habitats being connected, this is an important result to be highlighted. Even if it is lower, the difference in mean species richness is only 1-2 species on average, which again is a very important result. These nuances in what the data is telling are key and often missed throughout the paper. The questions also seems too scattered, with some focusing on big cats, while others are looking at the whole community. It is confusing and difficult to get what the real results and messages of the research are.

**Authors’ comment:** Thanks for this feedback. Given that our model was based in Bayesian inference, we didn’t feel the need to report a frequentist result of whether that difference in species richness was indeed “significant” or “not significant.” However, we do agree that this difference in species richness is biologically not that meaningful and that we should have expanded upon this in our discussion. Please note our expanded content in Lines. 385-388, 416-421, where we note that while these numbers may appear similar, when we subset those values to large prey species only, there is a bigger difference between JCUs and corridor. We also added the number of independent records for large species in the discussion to further illustrate this difference. In addition, we are happy to do a frequentist test of significance if requested by the reviewer, though for now we still stay with the spirit of the Bayesian framework. We made changes to the results and discussion section to simplify our results and highlight what we consider are the takeaway point of the manuscript.

Authors, please do not be discouraged by these comments. I congratulate you for this great effort. My comments are made in a constructive spirit, to bring out what I think could be an exceptional paper. I believe this research should be published, but the field design needs to be clarified, the questions need to be tightened and more focused, the process to reach the final model explicitly stated and the interpretation of the results put into a wider context. I have attached the MS with some additional comments and suggestions that I marked directly. The paper should also be reviewed by an English copy editor before resubmission to improve the style according to journal standards. Hope all this is useful.

**Authors’ comment:** Thank you for your support of our work and for your careful review of our manuscript -- we do indeed hope we can see this through to publication! We have also gone through your additional comments and suggestions directly in the manuscript and have unilaterally incorporated these into the revised manuscript. Thank you again.
