# Supplementary material for: Forest cover mediates large and medium-sized mammal occurrence in a critical link of the Mesoamerican Biological Corridor
Source: PLoS One. 2021 Mar 23;16(3):e0249072. doi: 10.1371/journal.pone.0249072 (PMC7996086; doi:10.1371/journal.pone.0249072)
Supplement: Attachment — Submitted filename: PlosONE Response to reviewers-D-20-25172_vMarch.docx [file pone.0249072.s008.docx]

**Original Manuscript ID:** PONE-D-20-25172

**Original Article Title: “**The status of medium and large-sized mammals in a critical link of the Mesoamerican Biological Corridor”

**To:** PLOS ONE Editorial Board

**Re:** Response to reviewers

To the Editorial Board at PLOS ONE,

Thank you for the effort you have spent in the second review of our manuscript, and for giving us the chance to address the point raised by one of the reviewers. You will note our responses in **bold** below.

We are sending 1) our response to reviewers’ comments (below), 2) a marked-up copy of our manuscript with tracked changes, and 3) an unmarked version of our revised paper without tracked changes.

Best regards,

___________________

Roberto Salom-Pérez

Costa Rica and Mesoamerica Director

Panthera

T. (506) 2234-8079/Cel. (506) 8538-4178

E. rsalom@panthera.org

6. Review Comments to the Author

Reviewer #1: Authors have addressed implemented all changes or provided valid justification to most comments. However, I still find an important conceptual problem related to my first observation in the first round. Bellow I copy my original comment to authors, their reply, and a new comment based on their response and the changes in the manuscript.

Original reviewer comment: “Authors are hypothesizing that presence of puma and jaguar is associated with large prey species richness. However, in the Introduction I did not observe a rationale that supports that hypothesis. Arguably, prey biomass is more important than richness to determine predator presence. For example, in Los Llanos of Venezuela, 70% of jaguar diet is made up of 4 species (2 peccaries, capybara and giant ant eater) (Polisar et al 2003); a similar case is observed by Aranda (1994) in Mexico and other studies. It will be helpful to expand the introduction to address this hypothesis.”

Response by authors: “This is a great point by the reviewer, and we can certainly see the argument that prey biomass is a core component determining presence of predators. However, it has also been found that the richness of large-bodied prey can be a major driver of predator presence at large scales (please see Petracca et al. 2018 in Journal of Applied Ecology for an example including all jaguar corridors within Central America). It was the strong effect of this covariate in the Petracca et al. 2018 paper that led us to include this covariate in our manuscript - we have added material in our Introduction (L. 105-109) to help support our hypothesis that prey richness can be a viable driver of predator occupancy.”


I am not convinced by the response of the reviewers for the following reasons:
1. Petracca et al. (2018) are not evaluating the occurrence of jaguar as a function of prey diversity. That study, which is based on interviews (i.e, no estimate on game abundance/biomass), finds that there is an association between prey diversity and jaguar presence. Authors need to recognize that correlation does not imply causation. For example, prey diversity can be positively associated with forest cover and in general, a good conservation status. Better conserved sites likely have higher abundance of game, including large bodied species, which actually is the pattern found in the current study; lines 384–388 in Discussion state that “…it is clear that biomass of game is determining the presence of jaguar and puma, not game diversity.”
2. In the new version of the manuscript, while talking about jaguar and puma, authors state that (lines 106–109) “…their presence has been associated with prey biomass and availability [22,54–56]. However, recent studies have also found that prey richness can have an effect on large carnivore presence or ecology (e.g., diet) [15,57].” Here authors cite Petracca et al. 2018 (15), which as stated above does not prove that prey richness has an effect on large carnivore presence. Also, reference 57 (Ferretti et al. 2020) evaluates the effect of prey richness on dietary breath of large predators (i.e, increased generalism); it does not evaluate the presence of large predators as a function of prey diversity. Authors should be careful in how they present their interpretation of others’ studies.
3. To test the hypothesis that prey richness influences large carnivore presence, other research approach needs to be implemented. Confounding factors such as habitat quality and prey biomass need to be controlled.

In summary, I think the manuscript would be clearer and more robust without objective 3: “investigate the relationship between large carnivore habitat use and prey richness”. Currently the discussion feels forceful and somewhat contradictory because authors try to include that pattern (prey diversity as a cause of presence) as conclusive. However, that pattern is not sustained by the authors own findings and research design (i.e., higher biomass in sites with higher diversity; comparison of better with less conserved areas). Objectives 1 and 2 are clear and supported by authors methodology and results, and are sufficient to make an interesting and solid paper.

**Authors´ response: Thank you very much for following up on this particular point in our manuscript.**

**Given your insightful observations above, we acknowledge that the wording we used may have led readers to incorrectly interpret the correlation we found between prey species richness and puma/jaguar occupancy as causation. Our aim was not to prove that there was a causative link between prey richness and carnivore occupancy but rather to test for an association, with a particular interest in differences in the associations of large versus medium-sized prey richness on carnivore occupancy. Our emphasis on an association between carnivore and prey in several forms, rather than a causation, can be found in L. 105-110.**

**We also agree that the association between prey richness and carnivore occupancy should not be a formal objective and have removed Objective 3 from our manuscript (paragraph starting L. 111). We also eliminated any reference to a causative link between prey richness and carnivore occupancy (L. 449-464), and clarify that additional work will be needed to determine if prey biomass or forest cover (rather than richness) are the true causative agents. After careful consideration, we decided to leave the correlation results in the manuscript, as they have merit for what they are (i.e., correlations) while also serving as a discussion point for further research.**

**Lastly, we have amended the title of our manuscript to focus on the strongest part of our manuscript (the community occupancy model): “Forest cover mediates** **large and medium-sized mammal occurrence in a critical link of the Mesoamerican Biological Corridor.”**
